# Local phylogenetic analysis identifies distinct trends in transmitted HIV drug resistance: implications for public health interventions

**DOI:** 10.1186/1471-2334-13-509

**Published:** 2013-10-30

**Authors:** James I Brooks, Harrison Niznick, Marianna Ofner, Harriet Merks, Jonathan B Angel

**Affiliations:** 1 National HIV & Retrovirology Laboratories, Public Health Agency of Canada, Ottawa, Canada; 2 Division of Infectious Disease, University of Ottawa, Ottawa, Canada; 3 Health Surveillance and Epidemiology Division, Public Health Agency of Canada, Ottawa, Canada; 4 Ottawa Hospital Research Institute, Ottawa, Canada

**Keywords:** HIV, Surveillance, Drug resistance, Molecular epidemiology, Clusters, Public health

## Abstract

**Background:**

HIV transmitted drug resistance (TDR) surveillance is usually conducted by sampling from a large population. However, overall TDR prevalence results may be inaccurate for many individual clinical setting. We analyzed HIV genotypes at a tertiary care setting in Ottawa, Ontario in order to evaluate local TDR patterns among sub-populations.

**Method:**

Genotyping reports were digitized from ART naïve patients followed at the Immunodeficiency Clinic at the Ottawa Hospital, between 2008 and 2010. Quality controlled, digitized sequence data were assessed for TDR using the Stanford HIV Database. Patient characteristics were analyzed according to TDR patterns. Finally, a phylogenetic tree was constructed to elucidate the observed pattern of HIV TDR.

**Results:**

Among the 155 clinic patients there was no statistically significantly difference in demographics as compared to the Ontario provincial HIV population. The clinic prevalence of TDR was 12.3%; however, in contrast to the data from Ontario, TDR patterns were inverted with a 21% prevalence among MSM and 5.5% among IDU. Furthermore, nearly 80% of the observed TDR was a D67N/K219Q pattern with 87% of these infections arising from a distinct phylogenetic cluster.

**Conclusions:**

Local patterns of TDR were distinct to what had been observed provincially. Phylogenetic analysis uncovered a cluster of related infections among MSM that appeared more likely to be recent infections. Results support a paradigm of routine local TDR surveillance to identify the sub-populations under care. Furthermore, the routine application of phylogenetic analysis in the TDR surveillance context provides insights into how best to target prevention strategies; and how to correctly measure outcomes.

## Background

HIV drug resistance (DR) compromises successful clinical outcomes in both treatment naïve and treatment experienced patients [1,2]. In the context of emergent HIV therapy such as occupational exposure, occult HIV DR may lead to failure of post-exposure prophylaxis [3,4]. For these reasons and also for the purposes of mapping the epidemic, population level surveillance of transmitted DR (TDR) remains important [5,6]; however, inherent in population analyses of TDR is the loss of resolution at the local level. True local HIV TDR prevalence is obscured through estimates based on larger data sets generated at the regional or national levels. For example, if two clinics with highly divergent HIV TDR prevalence’s are sampled, then when analyzed in aggregate, the average prevalence is inaccurate for both clinics. With new interventions such as treatment as prevention being considered, accurate local TDR data is critical for program implementation and for outcome measures.

HIV TDR was evaluated among anti-retroviral (ART) naïve patients referred over a period of 3 years to the Immunodeficiency Clinic in the 2nd largest city in Ontario, Canada. The Ottawa Hospital Immunodeficiency Clinic routinely carries out baseline HIV DR testing [7-9] which provided access to the genetic sequence of the virus from the clinical HIV genotype report. Using this information we re-interpreted the HIV genotype in order to determine the local TDR trends and analyzed the distribution of TDR within the sub-populations of the clinic cohort using molecular epidemiology.

## Methods

### Data collection

Clinical and demographic data were obtained via chart review for all treatment-naive HIV patients referred to the Ottawa Hospital Immunodeficiency Clinic from August 2007 until June 2010. Variables extracted from the chart included: date of birth, country of origin, date of initial HIV diagnosis, likely HIV exposure category likely date of infection according to recall, earliest available CD4 count, viral load, and history of previous anti-retroviral drug (ART) exposure. Exposure category for HIV infection was hierarchically categorized as follows: men who have sex with men (MSM), intravenous drug use (IDU), HIV endemic country, heterosexual contact, and other. Data were entered into a database that was then anonymized for analysis through the assigning of a random study number. Blood specimens were taken as part of standard care. This study was approved by the Ottawa Hospital Research Ethics Board.

### HIV genotyping

Plasma was separated from EDTA anticoagulated blood within 4 hours of collection and frozen at -80°C. Frozen plasma was shipped to the HIV Drug Resistance Testing Program at the BC Centre for Excellence in HIV/AIDS, Vancouver, British Columbia for genotyping. Hard copy Vircotype clinical HIV drug resistance reports that included the HIV *pol* sequence submitted for interpretation were returned to the clinician and placed on the chart.

### Digitization of nucleotide sequences

Nucleotide sequences from the Vircotype reports were digitized using Readiris Pro 11 optical character recognition (OCR) software (Iris Technologies, Delray Beach, Florida, USA). Digitized sequences were aligned and scanning errors corrected. To verify the fidelity of the scanning software, 10 digitized sequences were chosen at random and twice manually compared to the original Vircotype hard copies.

### Genotypic resistance analysis

155 quality controlled sequences were submitted in FASTA format to the Stanford HIV Database Calibrated Population Resistance tool (CPR) [10] in order to derive an unambiguous and stable measure of TDR [11]. TDR mutations were identified using the World Health Organization (WHO) surveillance drug resistance mutation list [12]. Integral quality control assessment of the CPR analysis revealed 12 further sequences with non-IUPAC letter codes that were corrected and those sequences were re-submitted.

### Phylogenetic analysis

Genotype sequences were edited, and aligned using Muscle [13], then trimmed to identical lengths (986 bp) within Geneious 5.4.3 [14]. 136 reference *pol* sequences from the Los Alamos HIV Sequence database [15] and 119 *pol* sequences from the Canadian national HIV drug resistance surveillance were aligned and trimmed as above. Codons that harboured TDR mutations identified in the CPR analysis were stripped from all of the sequences within the alignment in order to minimize convergent evolution artefact. A maximum likelihood tree was constructed using the general time reversible (GTR) model in PHYML [16] in order to determine phylogenetic interrelationships among viral sequences. Robustness of relationships among sequences was evaluated using bootstrap analysis with 100 replicates. Clusters having bootstrap values >98% and within cluster branch lengths of less than 0.04 were further evaluated [17]. The similarity of sequences within clusters was compared to background subtype B sequences using mean pair-wise distance within a Maximum Composite Likelihood model with pair-wise deletion [18].

### Statistical analysis

303 patients were referred to the Ottawa Hospital Immunodeficiency clinic between August 2007 and June 2010 for assessment and care of their HIV infection. 196 patients (65%) were ART naïve of which 162 had undergone baseline genotyping. 155 of the baseline genotype reports were successfully digitized and used in the subsequent analysis. Reasons for the unavailability of a genotype were: insufficient plasma (13); genotyping was performed elsewhere (10); viral load was too low (9); or genotype not ordered (2).

Statistical analysis was performed using EpiInfo 2000 [19]. Descriptive statistics included frequency analysis (percentages) for categorical variables and means for normally distributed continuous variables. To compare the means of the continuous data an unpaired t-test was used. Chi-square or Fisher’s exact probability tests were used for categorical data. To determine whether the HIV infected patients seen at the Ottawa Hospital clinic patients were similar to those seen in the province as whole, comparisons of the characteristics of our cohort were compared with those from all reported Ontario provincial HIV cases [20]. To evaluate the internal validity of the cohort, characteristics of patients with genotyping results were compared with those from all patients seen concurrently in the clinic. Odds Ratios (OR) and 95% Confidence Intervals (CI) were calculated. P-values less than 0.05 were regarded as statistically significant. Patients with incomplete data were excluded from the analysis.

## Results

### Sample characteristics

155 sequences from ART naïve subjects were analyzed. Of the 155 patients for whom diagnosis date was recorded (n=145), 75% were genotyped within 1 year of diagnosis. The mean elapsed time from patient’s recollection of diagnosis date, to genotype was 487 days, with a median of 56 days (IQR 234 days).

### Representativeness of the data

Epidemiologic characteristics of the 155 study cohort patients, for whom a genotype sequence was available, were compared with those from cases in the Report on HIV/AIDS in Ontario 2008 [20] and also to other clinic patients. There was no significant difference with respect to age at diagnosis, gender or risk factor for acquisition of HIV (Table 1) between either the study cohort or the patients in the clinic. Similarly, when compared to all other clinic patients, there were no significant differences among characteristics of patients for whom genotyping results were available.

**Table 1:** Comparison of HIV epidemiologic characteristics of study patients with provincial surveillance report

	**Ontario**^**1**^		**Ottawa**^**1**^		**Clinic Cohort (n=196)**		**Clinic Cohort with Genotyping (n=155)**	
	**No.**	**%**	**No.**	**%**	**No.**	**%**	**No.**	**%**
Gender								
M	7134	74.7	116	70.3	141	71.9	115	74.2
F	2411	25.3	49	29.7	55	28.1	40	25.8
Age at diagnosis								
0-19	634	2.1	N/A	N/A	6	3.1	5	3.2
20-29	7053	23.7	N/A	N/A	41	20.9	31	20.0
30-39	11251	37.8	N/A	N/A	73	37.2	61	39.4
40-49	6046	20.3	N/A	N/A	49	25.0	40	25.8
50<	2430	8.2	N/A	N/A	26	13.3	18	11.6
Unclear	2373	8	N/A	N/A	1	0.5	0	0.0
Exposure category								
MSM	4391	45.1	56	34.1	76	38.8	61	39.4
IDU	715	7.3	22	13.4	24	12.2	18	11.6
MSM/IDU	248	2.5	4	2.4	3	1.5	2	1.3
HIV-endemic	2236	23.0	54	32.9	44	22.4	34	21.9
Heterosexual	1825	18.7	25	15.2	44	22.1	35	22.6
Other	327	3.4	3	1.8	1	0.5	1	0.6
Unclear	0	0.0	0	0	4	2.0	4	2.6

N/A information not provided.

^1^*Source*: 2008 Report on HIV/AIDS in Ontario, Ontario HIV Epidemiologic Monitor Unit.

The mean age of patients in the study cohort was 37 years (median 36 years IQR (31, 44)) with 75% being male. The most commonly reported risk categories for HIV acquisition were MSM followed by birth in an HIV endemic country, with each accounting for approximately 1/3 of the group. Analyzed by gender, 58% of the men reported MSM as their risk factor for HIV infection, while heterosexual contact was the dominant risk factor among women (86%). The study cohort had an mean viral load of 4.17 log_10_ copies/ml (median 4.26 log_10_ copies/ml, IQR (3.67,4.72)). The mean CD4 count was 332 cells/ul (median 303.0 cells/ul IQR (149.5,473.5)). Sixty-eight percent of the study cohort was infected with subtype B virus with the next most common subtype being subtype C (23%).

### Drug resistance

Of the 155 ART-naïve subjects with digitized HIV pol sequence that were subjected to further analysis, OCR fidelity validation trials identified only 2 incorrect base identification errors out of 11,600 bases analyzed (99.98% concordance). Neither of these errors influenced the TDR interpretation.

The crude prevalence of any drug resistance for the period of 2007-2010 was 12.3%. Although the TDR prevalence appeared to increase over the study period this trend was not significant (Chi-square, p=0.65). Fifteen of the 19 cases of TDR were resistant to NRTIs only. Two patients had 2-class resistance with one having a combination of NNRT/NRTI resistance and the other patient exhibiting combined NRTI/PI resistance. One patient had resistance to all 3 classes of ART. Details of the patterns of TDR for each patient can be found in Table 2.

**Table 2:** Surveillance drug resistance mutations patterns for patients

	**TDR Mutations**		
**Patient**	**NRTI**	**NNRTI**	**PI**
1	D67N, K219Q		
2	D67N, K219Q		
3	K65K**R**, D67N, K219Q		
4		K103N	
5	D67N, K219Q		
6	D67N, K219Q		M46**L**M
7	D67N, K219Q		
8	D67N, K219Q		
9	D67N, K219Q		
10	T215C	K103N	
11	D67N, K70R, M184V, T215F, K219E	K103N	L90M
12	D67N, K219Q		
13	K70R		
14	D67N, K219Q		
15	D67N, K219Q		
16	D67N, K219Q		
17	D67N, K219Q		
18	D67N, K219Q		
19	D67N, K219Q		

Prevalence of each TDR mutation among the 155 specimens were: D67N (10.3%), K219Q (9.7%), K103N (1.9%), K70R (1.3%), with all others at 0.6%.

TDR was not evenly distributed within the sub-populations of the study cohort. Although 30% of the infections were non-B subtype, the prevalence of TDR was only 2% as compared to 17% among subtype-B infections. In addition, the prevalence of TDR among women was 2.5% versus 16% among the men. Consistent with the gender based distribution of drug resistance, the TDR prevalence was 21% in MSM and only 5.5% among IDU. Finally, there was a trend towards increased rates of TDR among patients who had genotyping within 6 month after diagnosis (Table 3).

**Table 3:** Comparison of patient characteristics between those with and without drug resistance

**Characteristic**	**Patients with TDR**	**Patients without TDR**	**OR (95% CI)**	**p-value**
	**Number (%)**	**Number (%)**		
	**N**=**19**	**N**=**136**		
Country of Birth				
Canada	12 (63.2%)	79 (58.1%)	1.44 (0.47, 4.62)	0.48
Other	7 (36.8%)	57 (41.9%)		
Gender				
Male	18 (94.7%)	97 (71.3%)	7.18 (1.06, 309.13)	0.03
Female	1 (5.3%)	39 (28.7%)		
Mean age (range)	38 (23-49)	37 (18-70)		0.73
Exposure Category				
MSM	13 (68.4%)	48 (35.3%)		
IDU	1 (5.3%)	17 (12.5%)		
MSM/IDU	1 (5.3%)	1 (0.7%)		
HIV endemic	2 (10.5%)	32 (23.5%)		
Heterosexual	0	35 (25.7%)		
Other	0	1 (0.7%)		
Unknown	2 (10.5%)	2 (1.5%)		
MSM	13 (68.4%)	48 (35.3%)	3.97 (1.30, 12.63)	0.005
All other	6 (31.6%)	88 (64.7%)		
Clade				
B	18(94.7%)	88 (64.7%)	9.73 (1.45,417.43)	0.009
Non-B	1 (5.3%)	48 (35.3%)		
CD4 Count				
<200	6 (31.6%)	41 (30.1%)		0.97
200-500	8 (42.1%)	64 (47.1%)		
>500	5 (26.3%)	29 (21.3%)		
Unknown	0	2 (1.5%)		
Viral Load				
<5,000	5 (21.1%)	36 (27.2%)		0.37
5,000-10,000	3 (15.8%)	13 (9.6%)		
10,000-100,000	8 (47.4%)	57 (41.2%)		
>100,000	2 (10.5%)	29 (21.3%)		
Unknown	1 (5.3%)	1 (0.7%)		
Time Between Diagnosis and Genotype				0.07*
<6 months	17 (89.5%)	88 (64.7%)		
6-12 months	0	7 (5.1%)		
>12 months	1 (5.3%)	34 (25.5%)		
Unknown	1 (5.3%)	7 (5.1%)		

* *Unknowns removed from analysis*.

* “*Zero*” *cell adjustments made in calculations*.

### Phylogenetic analysis

**Figure 1:**
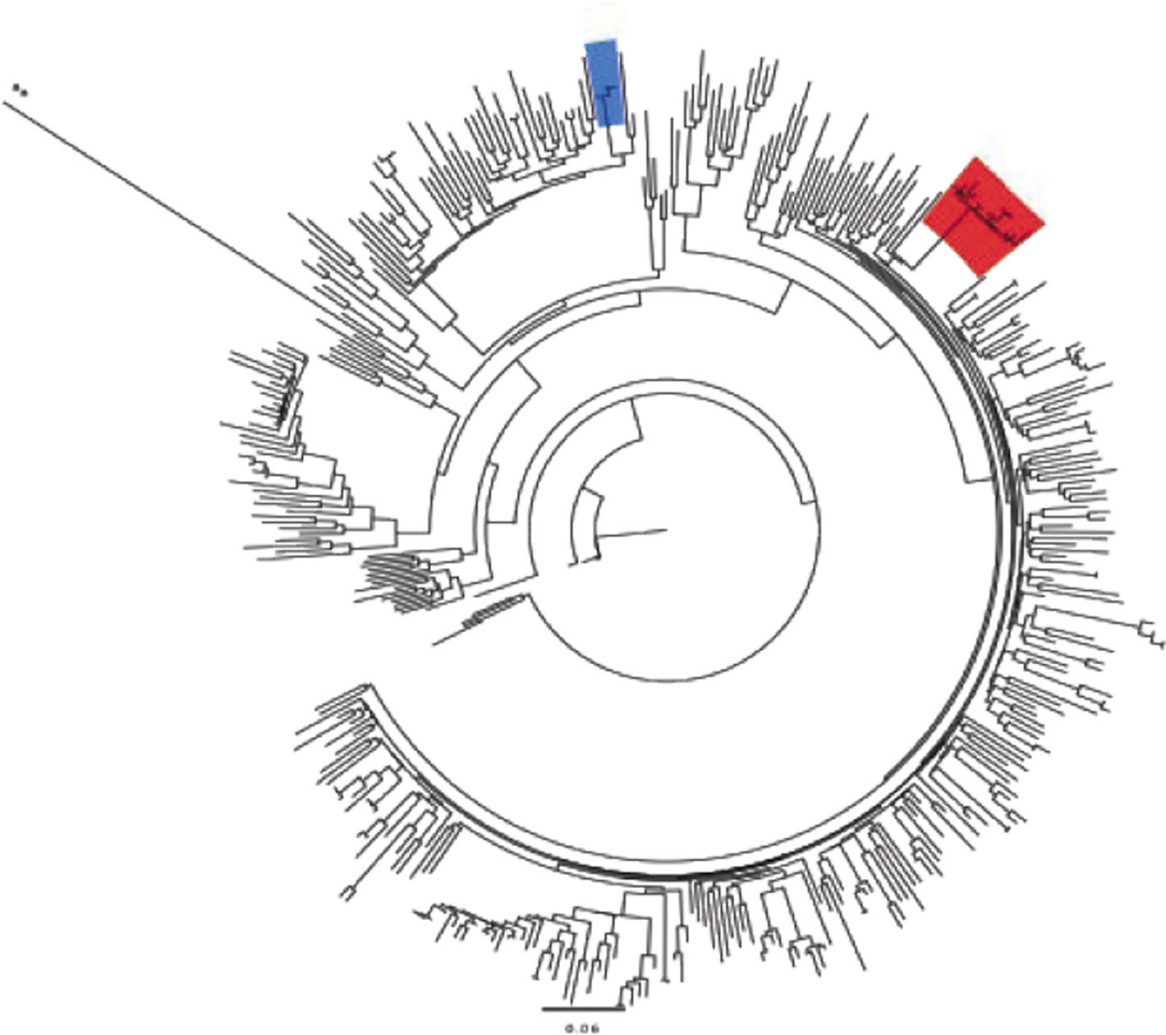
**Maximum likelihood tree of HIV pol sequences generated using GTR model in PHYML with 100 bootstrap replicates.** Sequences were trimmed to 986 bp with identified TDR mutation codons removed. 155 study cohort sequences are present in a background of 255 sequences further described in the methods. Orange box shows the D67N/K219Q cluster that was identified. For comparison purposes another cluster identified in the study cohort is identified in blue. ** Indicates the out-group N sequence N.CM.97.YBF106.

The most common pattern of drug resistance mutations seen was the combination of RT mutations D67N and K219Q conferring low-level resistance to AZT. In fact, 15 of the 19 drug resistant specimens collected over the three-year period contained this identical dug resistance pattern (Table 2) which warranted further investigation. A phylogenetic analysis using a GTR model in PHYML, established that 13 out of the 15 sequences containing the D67N/K219Q formed a distinct out-group with a bootstrap value of 100% (Figure 1). The monophyletic cluster possessed a within cluster pair-wise distance of less than 1%, compared with the background variation of 6%, which is consistent with HIV transmission over a shorter period of time. There were two additional D67N/K219Q containing sequences that were not found to be part of this cluster. These two sequences were not related to each other. 17 additional clusters were found within this cohort with most (60%) having only two members. There were 4 clusters with 3 members and 3 clusters with 4 members. No TDR was found within any of these other clusters (Figure 1).

The phylogenetically inferred relationships are strongly supported by the observed epidemiological characteristics. All of the patients with the D67N/K219Q resistance pattern are male, are infected with a clade B virus and either have MSM as their risk factor for acquisition or it is not reported (2 cases). These characteristics were significantly associated with this group (all OR > 6 and all p-values <0.02) when compared to all other genotyped patients. All but one of the cases with D67N/K219Q were less than 6 months induration, however the small numbers in the study prevented the correlation with recent infections being significant. The relationship between the pattern of clustering observed on the phylogenetic tree and the characterization of a clearly defined sub-population supports the interpretation of cluster identification as means to identify a group at risk of HIV infection.

## Discussion

HIV TDR Surveillance projects are typically designed to avoid sample bias by seeking out an ever enlarging (n) [5,21,22]. Paradoxically, the most relevant information for the clinician arrives from a biased but more relevant, local sample. Similar to other national surveillance projects, [23] ideal HIV TDR data should be systematically obtained at the local level, where it most accurately reflects the ecology of the virus, and then collated at a regional level. This approach would produce clinically relevant data and facilitate the population level study of HIV TDR dynamics [24,25]. This study is unique in that it compares the local viral HIV TDR landscape with that predicted to be present in a large population based study [26]. While the cohort of patients in this analysis was not significantly different with respect to age, gender, risk factors or ethnicity from the HIV infected population in Ontario; there were striking differences in the TDR distribution within our clinic sub-populations when compared to the province as a whole.

Initially appearing to be consistent with results from other groups [17,27-33], the average prevalence of TDR at 12.3% is heterogeneously distributed. TDR prevalence is lower in women, people with non subtype-B infections and acquisition of HIV through non-MSM contact [33,34]. However, in our cohort 1 in 5 patients who acquired their infection through sex with men harboured some form of TDR, almost double the average prevalence. More striking is that most recent available data from on Ontario-wide study revealed that TDR prevalences were three-fold higher among intravenous drug users (IDU) than among MSM [26]. Despite our clinic cohort appearing to be representative of the population of the province as whole, we observed the exact opposite pattern of TDR with MSM having four times the prevalence of TDR than was found among IDU. The distinct and contrasting TDR distribution observed among our clinic sub-populations was lost completely when data was analyzed at the provincial level highlighting the importance of local analysis of TDR patterns.

One of the significant drivers of the distribution of TDR, within our local sub-populations, was the cluster of related infections. Comparing the group of patients with the D67N/K219Q (n=15) with those patients with another pattern of TDR (n=4), it is clear that the epidemiologic characteristics of patients with TDR in our clinic were made up of those from this larger group. The D67N/K219Q combination has a low fitness cost to the virus, is durable in the recipient after infection, is readily transmitted and serves as an excellent marker for TDR [35]. The similar pattern of drug resistance found in more than three quarters of the resistant cases, cued the molecular epidemiology investigation that identified the cluster of related infections among MSM. However, in the absence of this TDR marker, a cluster of related viruses would go unrecognized and, along with that, the opportunity to recognize an outbreak. Phylogenetics are often applied to large data sets to illuminate transmission patterns or trends in drug resistance at the population level [36-42]. Our findings continue to support the value in applying routine phylogenetics at the clinical level to uncover related infections even in the absence of common patterns of TDR [41,42].

While having clinical implications, there is potential public health benefit in identifying a cluster of HIV transmission [43]. Although the limitations of phylogenetic analysis include the inability to attribute directionality of a transmission event [44,45], highly related viral sequences may correlate with shared social or risk-behaviour patterns [46]. If a cluster of related infections goes unrecognized by public health, an opportunity may be lost to mitigate ongoing transmission; especially originating from those individuals recently infected or from those not yet diagnosed. Public health education within the communities in which higher HIV TDR is found, more frequent testing of those within those communities and appropriate and early treatment are all public health interventions can reduce transmission and improve the outcomes within that community. From the outcome perspective, it is extremely important to understand the reasons for successes and failures of public health interventions. An occult transmission cluster, reflecting a negative result such as incident infection or drug resistance, can negatively bias the interpretation of a program that is actually effective. Studies are underway to examine how best to balance the use of phylogenetics in public health investigations with individual privacy [47].

There are some limitations to be considered in this study. Nearly 90% of the specimens that contained drug resistance were obtained less than 6 months after diagnosis while only 63% of the specimens without TDR were collected in the same timeframe. While our evaluation of TDR may be biased toward identifying less durable mutations the D67N/K219Q, which represented the majority of our TDR, has a low fitness cost and should be durable. The over-representation of these mutations among those recently diagnosed is likely consistent with the mutations acting as a marker for recent cluster formation. While we were diligent in identifying all new patients seen at the largest HIV referral center in Ottawa, there is the possibility of sample bias due to the unavailability of genotypes from some patients. Consequently, even though our cohort appears to be statistically representative of the epidemic in Ontario, our findings on TDR are valid only for the sub-populations seen at the Ottawa Hospital. This sample bias may also affect the depth of sampling in the phylogenetic analysis resulting in the possibility that the clustering of the D69N/K219Q mutations among MSM may be more widespread. Although our study possesses the theoretical limitations associated with sample bias, it is precisely these “limitations” that exposed the divergent TDR trends among our clinic sub-populations. Indeed, similar disparities in DR surveillance patterns have been observed in bacterial infections resulting in a similar call for local surveillance [48].

## Conclusion

We have shown that analysis of HIV genotypes, at the local level, reveals patterns of DR that are distinct from those described in surveillance reports encompassing larger geographic areas. Local molecular epidemiological analysis of these genotypes may provide insight into the reasons why these HIV DR patterns differ and expose occult infection clusters. Using the model for antibiotic resistance surveillance, collecting and analyzing HIV TDR data at the clinic level and then collating these results at provincial and national levels would both optimize patient care and provide more complete surveillance [23]. Finally, local phylogenetic analysis of clinical specimens may provide public health with additional tools for outbreak investigations.

## Competing interests

The authors declare that they have no competing interests.

## Authors’ contributions

JIB participated in the design of the study, designed data collection and HIV DR analysis, analyzed the data, performed the phylogenetic analysis, and wrote the manuscript. HN participated in the design of the project, conducted the chart review, gathered and verified data, performed preliminary data analysis and contributed to writing the manuscript. MO performed all the statistical analysis and contributed to writing the manuscript. HM organized data for analysis and verified data for accuracy. JBA participated in the design of the project, assisted with data collection and analysis, and contributed to writing the manuscript. Sequences are available in Genbank Accession numbers KF727690 - KF727963. All authors read and approved the final manuscript.

## Pre-publication history

The pre-publication history for this paper can be accessed here:

http://www.biomedcentral.com/1471-2334/13/509/prepub
